# A Tale of Two Emotions: The Diverging Salience and Health Consequences of Calmness and Excitement in Old Age

**DOI:** 10.1093/geroni/igaa057.2138

**Published:** 2020-12-16

**Authors:** Jeremy Hamm, Carsten Wrosch, Meaghan Barlow, Ute Kunzmann

**Affiliations:** 1 North Dakota State University, Fargo, North Dakota, United States; 2 Concordia University, Montreal, Quebec, Canada; 3 University of California, Berkeley, Berkeley, California, United States; 4 University of Leipzig, Leipzig, Sachsen, Germany

## Abstract

Using two studies, we examined the late life prevalence and health consequences of discrete positive emotions posited to motivate rest and recovery (calmness) or pursuit of novelty and stimulation (excitement). Study 1 assessed the salience of these discrete emotions in older adults (n=73, Mage=73) relative to younger adults (n=73, Mage=23) over a one-week period. Multilevel models showed that older (vs. younger) adults reported higher calmness and lower excitement. Study 2 examined the longitudinal health consequences of calmness and excitement in old age (n=336, Mage=75), as moderated by perceived control. Multilevel growth models showed that calmness, but not excitement, buffered against 10-year declines in psychological well-being (perceived stress, depressive symptoms) and physical health (physical symptoms, chronic conditions) for older adults with low perceived control. Results suggest that positive emotions with disparate motivational functions become more (calmness) or less (excitement) salient and have diverging implications for health in old age.

